# The role of ubiquitination in spinal and bulbar muscular atrophy

**DOI:** 10.3389/fnmol.2022.1020143

**Published:** 2022-10-06

**Authors:** Medha Sengupta, Anna Pluciennik, Diane E. Merry

**Affiliations:** Department of Biochemistry and Molecular Biology, Sidney Kimmel Medical College, Thomas Jefferson University, Philadelphia, PA, United States

**Keywords:** spinal and bulbar muscular atrophy, Kennedy’s disease, polyglutamine, androgen receptor, ubiquitin, proteasome, neurodegenerative

## Abstract

Spinal and bulbar muscular atrophy (SBMA) is a neurodegenerative and neuromuscular genetic disease caused by the expansion of a polyglutamine-encoding CAG tract in the androgen receptor (AR) gene. The AR is an important transcriptional regulator of the nuclear hormone receptor superfamily; its levels are regulated in many ways including by ubiquitin-dependent degradation. Ubiquitination is a post-translational modification (PTM) which plays a key role in both AR transcriptional activity and its degradation. Moreover, the ubiquitin-proteasome system (UPS) is a fundamental component of cellular functioning and has been implicated in diseases of protein misfolding and aggregation, including polyglutamine (polyQ) repeat expansion diseases such as Huntington’s disease and SBMA. In this review, we discuss the details of the UPS system, its functions and regulation, and the role of AR ubiquitination and UPS components in SBMA. We also discuss aspects of the UPS that may be manipulated for therapeutic effect in SBMA.

## Introduction

The ubiquitin-proteasome system (UPS) is of central importance to overall protein quality control in eukaryotic cells. It encompasses a vast, complex network of proteins that carry out a multitude of functions to preserve proteostasis. These functions include regulating intracellular protein levels by mediating their destruction, trafficking of proteins within and across subcellular compartments, regulating gene transcription, facilitating DNA damage repair, and mediating immune responses. Therefore, it is not surprising that dysfunction of UPS components has been implicated in diseases of neurodegeneration, viral infection, cancer, and other disorders (Rao et al., 2015; Budroni and Versteeg, 2021; Hwang et al., 2022; Park et al., 2022). The UPS also connects to the cell’s other protein degradation and quality control pathways, the unfolded protein response (UPR), endoplasmic reticulum-associated degradation (ERAD), and the autophagy-lysosome pathway (ALP), processes that also have been implicated in various neurodegenerative diseases (Chen et al., 2019; Lopata et al., 2020; Qu et al., 2021). The androgen receptor (AR), an important nuclear hormone receptor that plays a causative role in the neuromuscular disease spinal and bulbar muscular atrophy (SBMA), is regulated by the UPS for both its transcriptional functions and its degradation. This role of the UPS in AR function and metabolism, as well as its role in additional aspects of neuromuscular function in SBMA, is the focus of this review.

## Spinal and bulbar muscular atrophy

Spinal and bulbar muscular atrophy (SBMA; also known as Kennedy’s disease) is an adult-onset, slowly progressive, neuromuscular disease affecting 1 in 40,000 males (Fischbeck, 1997; Dejager et al., 2002; Pradat et al., 2020). It is one of nine polyglutamine (polyQ) repeat expansion neurodegenerative disorders that include Huntington’s disease (HD), dentatorubral-pallidoluysian atrophy, and the spinocerebellar ataxias 1, 2, 3, 6, 7, and 17 (Lieberman et al., 2019). An X-linked disease, SBMA is caused by expansion of a polyQ-encoding, polymorphic, CAG repeat within exon 1 of the AR gene to a number greater than 37 (La Spada et al., 1991).

SBMA primarily affects men (Müller et al., 2021); studies of cell and mouse models of SBMA reveal the requirement for circulating testosterone for the emergence and progression of disease symptoms (Katsuno et al., 2002, 2003; Takeyama et al., 2002; Chevalier-Larsen et al., 2004; Sopher et al., 2004; Yu et al., 2006). Patients experience facial, tongue, and proximal muscle weakness that is accompanied by cramps, intention tremor, and fasciculations (Dejager et al., 2002; Atsuta et al., 2006; Rhodes et al., 2009; Guber et al., 2018). Speech, swallowing, and mobility are progressively impaired (Guber et al., 2018), and many patients become wheelchair-bound (Atsuta et al., 2006). Aspiration pneumonia is a common cause of death for SBMA patients (Atsuta et al., 2006). In addition to neuromuscular symptoms, patients also experience signs of mild androgen insensitivity, as well as metabolic and sensory disturbances (Arnold and Merry, 2019).

Although SBMA patients frequently exhibit signs of partial androgen insensitivity, including gynecomastia, testicular atrophy, and infertility, such noted partial loss of AR function does not explain the mechanism for SBMA *neuromuscular* symptoms (Pinsky et al., 1992); rather, these symptoms in SBMA result primarily from mutant AR proteotoxicity (Merry et al., 1998; Katsuno et al., 2002). Nonetheless, it is possible that reduced AR transcriptional function in the neuromuscular system upon polyQ expansion may *exacerbate* the proteotoxic effects of mutant, misfolded, AR, since knockout of endogenous AR exacerbated symptoms in a mouse model of SBMA (Thomas et al., 2006). Moreover, AR is known to play a trophic role in motor neurons and an important anabolic role in muscle; both are the direct result of the transcriptional function of androgen-bound AR (Kurz et al., 1986; Marron et al., 2005; Fargo et al., 2008a; MacLean et al., 2008; Little et al., 2009). These positive AR functions in spinal motor neurons and muscle are reduced in SBMA mouse models, as evidenced by modified AR transcriptional functions in both motor neurons and muscle (Lieberman, 2018). The production in SBMA patients of only *mutant* AR protein, produced from this single mutant allele, results in AR protein with *characteristics of both proteotoxicity and partial loss of function*.

SBMA pathology is characterized by the loss of lower motor neurons (LMN) from the brainstem and anterior horn of the spinal cord in postmortem tissues (Sobue et al., 1989) and by muscle fiber atrophy, fiber-type grouping, and centralized nuclei (Sobue et al., 1989; Jokela et al., 2016), evidence of both neurogenic and myogenic causes. Moreover, the significant elevation of serum creatine kinase (CK) level (Chahin and Sorenson, 2009; Querin et al., 2016), implicates a primary involvement of muscle atrophy in disease pathogenesis (reviewed in: Arnold and Merry, 2019). In support of this idea, muscle tissue-specific deletion of polyQ-expanded AR alleviated signs of disease in animal models of SBMA, illustrating an important myogenic contribution to disease (Soraru et al., 2008; Cortes et al., 2014; Lieberman et al., 2014). Finally, muscle biomarkers in SBMA patients correlate more strongly with disease severity than do neuronal biomarkers (Lombardi et al., 2019).

In addition to these characteristic pathologies, affected SBMA tissues exhibit misfolding and predominantly, but not exclusively, nuclear aggregation of the mutant AR (Li et al., 1998a,b; Adachi et al., 2005; Suzuki et al., 2007). These neuronal intranuclear inclusions (NII) in patient tissue contain truncated N-terminal fragments of polyQ-expanded AR and ubiquitin (Li et al., 1998a,b, 2007; Heine et al., 2015). Additionally, chaperone proteins Hsp40, Hsp70, and Hsp90, and UPS components, including 20S proteasome, NEDD8, PA700, and REGγ, are detected in inclusions in cell and mouse models (Stenoien et al., 1999; Abel et al., 2001; Bailey et al., 2002; Yersak et al., 2017). P62 also accumulates in NII, as detected in patient tissue and a mouse model of SBMA (Doi et al., 2013).

Despite the fundamental knowledge of the genetic cause of this disorder, the growing understanding of the diverse symptom range and underlying molecular mechanisms, a number of well-characterized *in vitro* and *in vivo* disease models, and several potential interventions in clinical trials, there is still no effective treatment for SBMA (Arnold and Merry, 2019).

## The ubiquitin-proteasome system

### The proteasome

The UPS is responsible for the majority of intracellular protein degradation (Rock et al., 1994; Collins and Goldberg, 2017). The proteasome is a large (2.4 MDa), highly conserved, multisubunit protein complex with ATP-dependent proteolytic activity. It is found in both the nuclear and cytosolic compartments of eukaryotic cells. It consists of a cylindrical 20S core particle that can be found either unbound as a free 20S proteasome, or in complex with a regulatory particle flanking it on either or both sides. The constitutive 26S proteasome is comprised of a 20S core particle bound to a single 19S regulatory particle (19S–20S), while the 30S proteasome contains two 19S regulatory particles, one on either side (19S-20S-19S) (Coux et al., 1996; Baumeister et al., 1998; Demartino and Gillette, 2007). Although not bound by any regulatory particle, the free 20S proteasome is also capable of degrading polypeptides, particularly during periods of cellular stress (Raynes et al., 2016).

The 20S core particle of the proteasome is composed of 4 stacked rings, two outer α-rings and two catalytic inner β-rings, each comprised of 7 subunits. Located in the inner β-rings are the three active sites, each with distinct amino acid cleavage specificity and each bearing an active site threonine amino acid. The β1 caspase-like subunit cleaves after acidic sites, the β2 trypsin-like subunit cleaves after basic residues, and the β5 chymotrypsin-like subunit cleaves after large hydrophobic sites (Wilk and Orlowski, 1983; Voges et al., 1999).

The regulatory particle of the proteasome recognizes and binds polyubiquitin-tagged substrate proteins, cleaves the ubiquitin chain, unfolds the protein in an ATP-dependent manner, and feeds the polypeptide into the center of the 20S core. Within the 20S core particle, the movement-coupled proteolysis occurs in an ATP-dependent manner. The regulatory particles in an assembled proteasome complex determine its structure and function, resulting in distinct proteasome types (Collins and Goldberg, 2017).

### Ubiquitination

Ubiquitination is a post-translational modification (PTM) wherein a single ubiquitin protein (monoubiquitination) or a chain of multiple, linked ubiquitin proteins (polyubiquitination) is covalently attached to a substrate protein and alters its cellular fate (Hershko, 1988). Ubiquitin is a highly conserved and very small (8.6 kDa) protein, found “ubiquitously” in all cell types. Mammals have four distinct genes (*UBB*, *UBC*, *UBA52*, and *RPS27A*) encoding ubiquitin precursor proteins. The ubiquitin precursors are polymeric proteins which are cleaved by specific deubiquitinases to produce free monoubiquitin (Finley et al., 1989; Grou et al., 2015). *UBB* and *UBC* code for polyubiquitin (Lund et al., 1985; Wiborg et al., 1985) while *UBA52* and *RPS27A* code for fusion proteins consisting of a single ubiquitin and a ribosomal protein, L40 and S27a, respectively (Redman and Rechsteiner, 1989; Baker and Board, 1991).

Three classes of enzymes work in concert to mediate protein ubiquitination in a multi-step process. First, an ATP-dependent E1 activating enzyme forms a thioester bond with the C-terminus of a free ubiquitin. Next, the activated ubiquitin is transferred to the active cysteine site of an E2 conjugating enzyme. In the third and final step, an E3 ubiquitin ligase catalyzes the ubiquitin transfer to a substrate protein, forming an isopeptide bond (Hershko, 1988; Ciechanover, 1998). This process can occur once for monoubiquitin, or multiple times sequentially to result in a polyubiquitin chain. A ubiquitin chain is typically attached to lysine residues on substrate proteins; however, non-lysine ubiquitination on cysteine, serine, and threonine residues, and at the N-terminus of substrate proteins has also been documented (McDowell and Philpott, 2016; McClellan et al., 2019).

### E3 ubiquitin ligases

There are two known human E1 activating enzymes: Ube1 and Uba6 (Schulman and Wade Harper, 2009; reviewed in: Wang and Zhao, 2019), approximately 40 known human E2 conjugating enzymes (Michelle et al., 2009; Wenzel et al., 2011b), and over 600 known human E3 ubiquitin ligases (Li et al., 2008; Medvar et al., 2016). Additionally, E3/E4 elongation ligases are capable of adding polyubiquitin chains *en bloc* (Koegl et al., 1999). E3 ubiquitin ligases represent an important layer of substrate specificity for ubiquitination. They are classified into four main structurally and mechanistically distinct families (Buetow and Huang, 2016; Morreale and Walden, 2016; Zheng and Shabek, 2017; Yang et al., 2021).

Members of the **HECT** (Homologous to the E6-AP Carboxyl Terminus) family of ubiquitin E3 ligases possess a HECT domain in the C-terminus and ubiquitinate substrate proteins in a two-step process. The N-terminus of the HECT E3 ligase binds both the substrate protein and the ubiquitin-bearing E2-conjugating enzyme. In the first step of ubiquitination, a ubiquitin is transferred from the E2 enzyme to a catalytic cysteine residue in the conserved C-terminal HECT domain. In the next step, ubiquitin is transferred from the cysteine site to the substrate protein (Huibregtse et al., 1995). Based on N-terminal structural differences that impart specificity, the HECT family can be further divided into the Nedd4, HERC, and “other HECT” E3 ligase sub-families (Rotin and Kumar, 2009; Weber et al., 2019).

The **RING** (Really Interesting New Gene) E3 ubiquitin ligases, the largest family of E3 ligases, contain an N-terminal RING domain that binds the E2 conjugating enzyme. Ubiquitination takes place in a single-step reaction, with ubiquitin being transferred directly from the E2 enzyme to the substrate protein. The RING E3 ligase family can be further divided based on functional structure, i.e., monomeric, dimeric (for example, RNF4), multisubunit complex. The multiprotein RING E3 ligases include the cullin-RING ligase (CRLs) protein complexes. These are composed of a RING-protein, an adaptor protein, and a substrate receptor protein. Some multisubunit complex RING E3 ligases can be very large, such as the APC/C (Anaphase Promoting Complex/Cyclosome) which can be comprised of 19 proteins, and the SCF (Skp1, Cullin1, and F-box) E3 ligase complexes (Chang and Barford, 2014; Metzger et al., 2014; Buetow and Huang, 2016; Yang et al., 2021).

The U-box and RBR E3 ubiquitin ligases are the two smaller families of E3 ligases. **U-box** E3 ligases contain a conserved C-terminal U-box domain that is structurally similar to a RING domain (Aravind and Koonin, 2000; Hatakeyama et al., 2001). In addition to E3 ligase activity, they are capable of E4 elongation activity; that is, they work in conjunction with another E3 ligase to add polyubiquitin chains to an existing ubiquitin chain. U-box E3 ligases include E4B (also known as UBE4A) (Koegl et al., 1999) and CHIP (Imai et al., 2002).

The **RBR (RING-Between-RING)** E3 ligases contain two RING domains separated by an IBR (in-between-RING) domain (Aguilera et al., 2000). RBR E3 ligases bind to the activated E2 enzyme *via* the RING1 domain. Ubiquitination is a two-step reaction. In the initial step, ubiquitin is transferred to a catalytic RING2 domain; in the next and final step, ubiquitin is transferred from the RING2 domain to the substrate protein. RBR E3 ubiquitin ligases include Parkin, the E3 ligase implicated in Parkinson’s disease (Wenzel et al., 2011a).

### Deubiquitinases

Deubiquitinases (DUBs) are isopeptidases that hydrolyze the amide bond between a ubiquitinated protein and its ubiquitin molecule or chain, and thus catalyze the removal of ubiquitin from substrate proteins. In the cell, DUBs can be found either associated with or independent of the proteasome. Proteasome-independent DUBs modify the intracellular fate of ubiquitinated substrate proteins and provide an additional layer of specificity and regulation of ubiquitination (Komander and Rape, 2012).

Six structurally distinct families of deubiquitinating enzymes have been identified; these include four cysteine protease DUBs, the zinc metalloprotease DUBs, and the motif-interacting-with-ubiquitin (MIU)–containing DUB family (MINDYs). The four cysteine protease DUB families include the ubiquitin-specific proteases (USP), the ubiquitin C-terminal hydrolases (UCH), the ovarian tumor proteases (OTU), and the Machado-Joseph disease protein domain proteases (MJD or Josephin). The largest and best studied of the DUB protein families is the USP family (Amerik and Hochstrasser, 2004; Mevissen and Komander, 2017).

In addition to having substrate protein specificity, some DUBs exhibit preferences for cleaving certain linkage types (Mevissen and Komander, 2017). Moreover, DUBs may work in tandem with an E3 ligase to exert opposing effects on a substrate protein to regulate its abundance or subcellular localization (Kim and Sixma, 2017; Mevissen and Komander, 2017). For example, the intricate mutual associations of the DUB USP7, its substrate protein p53, and the E3 ligase MDM2 have been well-studied. Tumor suppressor p53 is a transcription factor involved in cell cycle arrest and apoptosis, among other cellular functions (Vogelstein et al., 2000). MDM2 targets it for proteasomal degradation, maintaining a relatively short half-life of p53 (Maki et al., 1996). The DUB USP7 is capable of binding and deubiquitinating p53 or MDM2, thereby protecting them from proteasomal degradation. USP7 has greater binding affinity for MDM2 than for p53 (Hu et al., 2006; Sheng et al., 2006), and at cellular homeostasis, it deubiquitinates and stabilizes MDM2 (Brooks et al., 2007). However, during periods of cellular stress, USP7 preferentially binds p53, protecting it from degradation. Additionally, USP7-MDM2-p53 form a multiprotein complex (Brooks et al., 2007). Other examples of DUB-E3 ligase interactions and tandem function on the same substrate protein include USP39/TRIM26 (Li et al., 2021) and USP18/Skp2 (Tan et al., 2018).

### Functions of the ubiquitin-proteasome system

The intracellular functions of the UPS can be broadly categorized as proteolytic or non-proteolytic. To a great extent, these functions depend on the ubiquitin code—a large diversity of ubiquitin chain and linkage types associated with different cellular fates. For many years, the role of polyubiquitin modification in regulating proteasomal degradation was thought to be solely due to K48-linkages, with all other chain types serving a non-proteolytic role. However, the ubiquitin code is revealing itself to be far more versatile and complex than was initially believed. A thorough review of the cellular processes associated with distinct ubiquitin linkages was recently published (Blount et al., 2020); only a short overview is thus provided here.

Polyubiquitin chains consist of ubiquitin proteins linked *via* one of seven lysines (K6, K11, K27, K29, K33, K48, K63) (Peng et al., 2003) or its N-terminal methionine residue (M1). Polyubiquitin chains can be homotypic, i.e., made up of only one linkage type, or heterotypic/mixed (Mevissen and Komander, 2017). Proteins may also be modified by monoubiquitin; this modification is associated with protein trafficking, including endocytosis (Marmor and Yarden, 2004) and nuclear export (Li et al., 2003), histone modification and regulation of transcriptional activity (Zhang, 2003), and more recently, with proteasomal degradation (Braten et al., 2016; Livneh et al., 2017).

The **K48-linked** tetraubiquitin chain topology is the most abundant and is associated with degradation mediated by the 26S proteasome (Chau et al., 1989; Gregori et al., 1990; Peng et al., 2003). However, a notable exception is found in the K48-ubiquitination of Met4, which regulates its transcriptional activity (Flick et al., 2004) in the absence of degradation. The ubiquitin code is further complicated in that, along with monoubiquitin and K48 linkages, K11, K29, and K63-linked polyubiquitin chains can also serve as degradation signals (Komander and Rape, 2012). It is also important to note that the proteasome can mediate protein degradation in a ubiquitin-independent manner (Driscoll and Goldberg, 1989; Buneeva and Medvedev, 2018).

Non-proteolytic roles for polyubiquitination include, for **K6** linkages, mitochondrial homeostasis (Durcan et al., 2014) and the DNA damage response (Wu-Baer et al., 2003; Elia et al., 2015; Blount et al., 2020), and for **K27** linkages, cellular proliferation (Shearer et al., 2022) and the innate immune response (Yin et al., 2019). **K29** polyubiquitin linkages play a role in the cellular stress response and in regulating the cell cycle (Yu et al., 2021) while **K33-linked** chains are involved in T cell activation and signaling (Huang et al., 2010; Yang et al., 2015). **K63-linked** polyubiquitin chains play important roles in intracellular trafficking (Lauwers et al., 2009; Erpapazoglou et al., 2014), cell signaling (Deng et al., 2000; Wang et al., 2001), and DNA damage repair (Liu et al., 2018). They are also associated with autophagic targeting, and particularly with the selective autophagy of aggregates (aggrephagy) (Olzmann et al., 2007; Olzmann and Chin, 2008; Tan et al., 2008). Finally, **linear polyubiquitin** chains are unique in that, instead of linking *via* a lysine residue of ubiquitin, they consist of a ubiquitin protein linked to the C-terminal glycine of another ubiquitin at its N-terminal methionine (M1) (Kirisako et al., 2006). They are formed by the E3 ligase linear ubiquitin chain assembly complex (LUBAC) and play a role in NF-kappaB activation and immune signaling (Shimizu et al., 2015).

### Regulation of the ubiquitin-proteasome system

Multiple layers of regulation of both degradative and non-degradative ubiquitination have been discovered. Many of these mechanisms are substrate-specific. E3 ubiquitin ligases and DUBs contain specific substrate recognition domains. Additionally, substrate proteins may contain general recognition signals for components of the UPS. Degrons or degradative signals are short amino acid sequences on substrate proteins associated with ubiquitination and proteasomal degradation. Both N-terminal and C-terminal degrons are well-studied (Varshavsky, 2008, 2019). Some proteins that harbor a PEST sequence—an amino acid sequence enriched in proline (P), glutamic acid (E), serine (S), and threonine (T)—have been found to undergo rapid degradation (Rogers et al., 1986). Intrinsically disordered regions of proteins represent targeting domains for proteasomal degradation (Guharoy et al., 2016). Additionally, misfolded proteins are specifically ubiquitinated and then degraded by the UPS or ALP (Amm et al., 2014).

PTMs play a critical role in UPS regulation by affecting the ubiquitination status of substrate proteins. E3 ligases and DUBs are also subject to regulation by PTMs. Finally, PTMs on ubiquitin itself can impact polyubiquitin chain formation; for example, ubiquitin acetylation inhibits chain elongation (Ohtake et al., 2015; Song and Luo, 2019). Importantly, many E3 ligases are capable of autoubiquitination, and thus negatively regulate their own intracellular levels (De Bie and Ciechanover, 2011).

Subcellular compartmentalization of substrate proteins and of the different UPS components also contributes to the regulation of the UPS. Some DUBs are predominantly nuclear or cytosolic, while others localize in both compartments. This impacts their ability to deubiquitinate particular substrate proteins based on their localization (Urbé et al., 2012). Similarly, E3 ligases are affected by localization as well (Checquolo et al., 2010; Breckel and Hochstrasser, 2021). Additionally, some E3 ligases localize primarily at the endoplasmic reticulum (ER) and are involved in ER-associated degradation (ERAD), while others are associated with Golgi bodies, the cell membrane, or mitochondria and peroxisomes, as reviewed by Rusilowicz-Jones et al. (2021).

Finally, ubiquitin-like proteins include SUMO (small ubiquitin-related modifier) and NEDD8 (neural precursor cell expressed, developmentally downregulated 8). In addition to modifying substrate proteins, they can also modify existing polyubiquitin chains (Herrmann et al., 2007). Other factors impacting protein ubiquitination, like the maintenance of the free ubiquitin pool and transcriptional regulation of ubiquitin genes are detailed by Kimura and Tanaka (2010).

## Androgen receptor lifecycle and ubiquitination of androgen receptor

### Androgen receptor

In order to understand the role of ubiquitination in SBMA, a discussion of wild-type AR (WT AR) is warranted. The AR gene (AR; gene: NR3C4) that encodes the 920-amino acid, 110 kDa protein, is located on the X chromosome at Xq11.2-Xq12 (Brown et al., 1989). Its primary physiological ligands are testosterone and its metabolite 5α-dihydrotestosterone (DHT) (Georget et al., 1997). AR is widely expressed in mammalian tissues, including in the central and peripheral nervous systems, muscle, and male reproductive organs (Rana et al., 2014). A transcription factor, it controls the expression of a multitude of target genes (Jia et al., 2008; Jiang et al., 2009; Massie et al., 2011; Jin et al., 2013), including genes responsible for embryonic sex differentiation and development of male secondary sex characteristics (Quigley et al., 1995; Hiort, 2013). AR and androgens play important roles in neurons, including during development and in response to injury (Pérez and Kelley, 1996; Fargo et al., 2008b; Davey et al., 2017; Mhaouty-Kodja, 2018). In skeletal muscle, AR and androgens play an important anabolic role, including effects on muscle mass and strength (Sheffield-Moore et al., 1999; Chen et al., 2005; Kadi, 2008; Leblanc et al., 2011; Davey et al., 2017).

The AR is composed of four primary domains: the intrinsically disordered N-terminal domain (NTD) (Lavery and McEwan, 2008; Sheikhhassani et al., 2022), a DNA-binding domain containing two zinc fingers (Shaffer et al., 2004), a hinge region, and a C-terminal ligand-binding domain (LBD) (Brinkmann et al., 1989). The AR contains three transactivation domains, of which the most potent is the AF1 in the NTD (Christiaens et al., 2002). The other transactivation domains, AF2 and BF3, are located in the LBD and are only exposed upon ligand-binding (He et al., 2004b; Estébanez-Perpiñá et al., 2007; Buzon et al., 2012). AR translocation to the nucleus is facilitated by a bipartite nuclear localization signal (NLS) within the hinge region (Zhou et al., 1994). Nuclear export may be regulated by a nuclear export signal (NES*^AR^*) in the carboxyl-terminal region (amino acids 744-818) (Saporita et al., 2003; Gong et al., 2012) although a specific NES has not been defined. The polymorphic glutamine repeat is located in the NTD (La Spada et al., 1991).

### Lifecycle of wild-type AR

A steroid hormone nuclear receptor, AR exists in the cytoplasm in an inactive and monomeric form in a multiprotein aporeceptor complex along with p23, immunophilins, and chaperone proteins such as Hsp70, Hsp40, and Hsp90. Ligand-binding induces a conformational change and reorganization of the aporeceptor complex (Ni et al., 2013) and induces the interaction between the N-terminal FQNLF motif and AF2 domain in an intramolecular N/C interaction (He et al., 2004b). Ligand-binding induces dissociation of importin 7 (Ni et al., 2013) and chaperone proteins Hsp70 and Hsp40 (Eftekharzadeh et al., 2019). The intramolecular N/C interaction exposes the hinge region NLS, allowing α-importin to bind AR and facilitate nuclear translocation (Zhou et al., 1994; Cutress et al., 2008; van Royen et al., 2012).

Within the nucleus, AR forms homodimers on DNA (Langley et al., 1995; El Kharraz et al., 2021). While both monomeric and dimeric AR are found in the nucleus (van Royen et al., 2012), it is the dimeric AR that predominantly interacts with DNA at androgen response elements (AREs) in promoter or enhancer regions of target genes and recruits coregulatory proteins (Wong et al., 1993; Shaffer et al., 2004). There, N/C interactions and LBD interactions facilitate AR dimerization in a unique head-to-head and tail-to-tail conformation, revealed by cryo-EM (Yu et al., 2020). AR assembles a transcriptional complex containing co-regulators, RNA polymerase II, and histone-modifying enzymes (Chang and McDonnell, 2005; Yu et al., 2020). Over 270 co-activators and co-repressors of AR have been identified (DePriest et al., 2016), including steroid receptor coactivator (SRC) family proteins (Bevan et al., 1999), CREB-binding protein (CBP) (Aarnisalo et al., 1998), p300 (Zhong et al., 2014), and UBC9 (Poukka et al., 1999).

Details of AR nuclear export are lacking; however, it is known that the wild-type AR is primarily degraded in the cytoplasm by the 26S proteasome (Sheflin et al., 2000; Gong et al., 2012).

### E3 ligases mediating ubiquitination of wild-type AR

**FIGURE 1:**
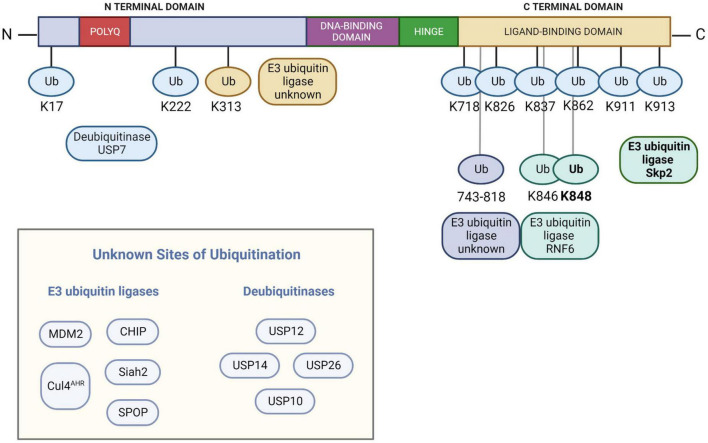
The known E3 ubiquitin ligases and deubiquitinases that impact the ubiquitination status of the androgen receptor, along with sites of ubiquitin modification (if known). Note that Skp2 modifies K848 with K48 polyubiquitin chains while RNF2 modifies both K846 and K848 with K6, K27, and K63 polyubiquitin chains. AR numbering is based on NCBI reference sequence NM_000044.6 and UniProtKB/Swiss-Prot: P10275.3. Figure created with BioRender.com.

A number of proteins are involved in regulating the ubiquitination status of AR. The functions of these proteins are not solely degradative; both proteolytic and non-proteolytic ubiquitinating activities have been identified. These activities regulate AR stability, intracellular localization, and transactivation (Jaworski, 2006). An overview of the E3 ubiquitin ligases and DUBs known to act on AR and alter its ubiquitination state is shown in Figure 1.

Several E3 ubiquitin ligases have been described that modify WT AR. The best-studied E3 ubiquitin ligase acting on AR is the U-Box protein COOH terminus of the Hsp70-interacting protein (CHIP). CHIP polyubiquitinates AR and targets it for proteasomal degradation (He et al., 2004a; Sarkar et al., 2017). CHIP-mediated ubiquitination requires the presence of chaperone proteins such as Hsp90 or Hsp70 and Hsp40 (Murata et al., 2001; Wang et al., 2010; Chymkowitch et al., 2011). Since CHIP predominantly interacts with cytoplasmic AR (He et al., 2004a), it is likely to be at least partly responsible for the reduced stabilization of AR in the absence of hormone (Kemppainen et al., 1992).

RING-finger E3 ubiquitin ligase MDM2 polyubiquitinates AR and mediates its degradation (Lin et al., 2002; Xu et al., 2009; Vummidi Giridhar et al., 2019). Its actions on AR are dependent on Akt-regulated phosphorylation (Lin et al., 2002). MDM2 acts on AR while it is bound to DNA (Lv et al., 2021). In addition to polyubiquitinating AR and targeting it for proteasomal degradation, MDM2 may monoubiquitinate DNA-bound AR and enhance its transcriptional activity (Gaughan et al., 2005).

Other proteins that polyubiquitinate AR and target it for proteasomal degradation have been identified. The cullin 3 (CUL3)-based E3 ligase SPOP polyubiquitinates AR in a manner that is regulated by S648 and T649 in the hinge region (An et al., 2014). In addition to targeting AR for proteasomal degradation (Khan et al., 2006; Rees et al., 2006; Chen et al., 2022), SPOP also polyubiquitinates and mediates the degradation of AR co-regulator SRC-3 (Li et al., 2011; Geng et al., 2014), thus impacting AR transcriptional activity in two ways. Siah2 adds K48-linked polyubiquitin chains to regulate the level of transcriptionally inactive AR (NCORC1-bound AR) (Qi et al., 2013; Jing et al., 2018). Skp2 polyubiquitinates AR at K848 (Li et al., 2014) and targets it for proteasomal degradation (Li et al., 2014; Chen et al., 2022). AHR acts on AR as part of a CUL4 complex (Ohtake et al., 2007, 2009). Exported AR is polyubiquitinated at the NES^AR^ and targeted for proteasomal degradation (Gong et al., 2012), although the E3 ligase involved in this ubiquitination is unknown.

Other E3 ligases acting on AR directly impact AR transactivation. E3 ligase RNF6 polyubiquitinates AR at K846 and K848. Unlike the role of Skp2, however, RNF6 promotes AR transcriptional activity on specific target genes *via* K6, K27, and K63 linkages and appears to have no impact on AR degradation (Xu et al., 2009; Sarkar et al., 2014; Lim et al., 2017). Ubiquitination at K313 also facilitates AR transactivation (McClurg et al., 2017).

### Deubiquitinases mediating deubiquitination of wild-type AR

Several cysteine protease DUBs have been reported to cleave ubiquitin from AR. USP10 deubiquitinates DNA-bound AR and promotes its transcriptional activity (Faus et al., 2005). USP12 (Burska et al., 2013; McClurg et al., 2014) and the proteasome-associated DUB USP14 (Liao et al., 2017, 2018; Xia et al., 2019) deubiquitinate and stabilize AR. USP7 deubiquitinates AR on AREs on AR target genes, stabilizing it and promoting its transcriptional activity (Chen et al., 2015). USP26 deubiquitinates nuclear AR, abrogating the ubiquitinating effects of MDM2 (Dirac and Bernards, 2010; Wang et al., 2020). Notably, both USP26 (Lahav-Baratz et al., 2017) and USP7 (Marine et al., 2006) also exert their deubiquitinating activity on MDM2.

### Regulation of androgen receptor ubiquitination

PTMs such as phosphorylation and acetylation are involved in the regulation of ubiquitination of AR for proteasomal degradation. Phosphorylation, in particular, has a pivotal role in the selection of E3 ligases. Phosphorylation driven by the kinase Akt at S215, S516, and S792 (Lin et al., 2001, 2002; Palazzolo et al., 2007, 2009; Chymkowitch et al., 2011) plays a role in MDM2-mediated ubiquitination and subsequent AR degradation. Phosphorylation by PAK6 at S579 also facilitates MDM2-mediated ubiquitination and proteasomal degradation (Liu et al., 2013).

CHIP interacts with and ubiquitinates AR in both a phosphorylation-dependent (Rees et al., 2006) and phosphorylation-independent manner (He et al., 2004a; Chymkowitch et al., 2011). Another report suggests that the activity of MDM2 or CHIP on AR is dependent on AR phosphorylation status (Chymkowitch et al., 2011). Additionally, the chaperone protein Hsp70 and its co-chaperone Hsp40 are important for CHIP-mediated ubiquitination of Hsp90 client proteins like AR (Wang et al., 2010; Eftekharzadeh et al., 2019). On the other hand, p300-mediated acetylation of AR prevents its ubiquitination and degradation, and promotes its transcriptional activity (Zhong et al., 2014). Other UPS components regulate AR activity and/or levels, although the mechanistic details of their roles have not been elucidated.

### Polyglutamine-expanded androgen receptor and spinal and bulbar muscular atrophy

The discovery of the critical role for AR hormone binding in SBMA led to an understanding of the role of specific hormone-dependent aspects of AR metabolism in disease. Nuclear localization of the mutant AR is required for its toxicity (Montie et al., 2009; Nedelsky et al., 2010); retention of the AR in the cytoplasm promotes its autophagic degradation and reduces its toxicity (Montie and Merry, 2009; Montie et al., 2009). On the other hand, promoting the nuclear export of polyQ-expanded AR led to enhanced degradation *via* the proteasome (Arnold et al., 2019). In addition, the N/C interaction is required for disease, both *in vitro* (Orr et al., 2010) and *in vivo* (Zboray et al., 2015). Inhibiting the N/C interaction promoted phosphorylation at S16, a PTM required for this neuroprotection (Zboray et al., 2015). Moreover, both AR arginine methylation (Scaramuzzino et al., 2015) and AR acetylation (Montie et al., 2011) represent PTMs of the mutant AR that are required for toxicity; in addition, roles for NLK and CK2 in modulating both the phosphorylation and toxicity of mutant AR indicate a role for AR phosphorylation in SBMA (Todd et al., 2015; Polanco et al., 2016). PolyQ-expanded AR exhibits increased DNA-binding (Belikov et al., 2015) and both increased and decreased transcriptional activity compared to WT AR (Mhatre et al., 1993; Kazemi-Esfarjani et al., 1995; Choong et al., 1996; Nakajima et al., 1996; Irvine, 2000; Lieberman et al., 2002; Sheppard et al., 2011; Belikov et al., 2015; Scaramuzzino et al., 2015; Badders et al., 2018). PolyQ-expanded AR also exhibits differential protein interactions vs. WT AR (Pluciennik et al., 2021), including its interactions with UPS components USP26 and USP7.

## The role of polyglutamine-expanded androgen receptor ubiquitination and ubiquitin-proteasome system components in spinal and bulbar muscular atrophy

Not only is the wild-type AR primarily degraded by the proteasome (Sheflin et al., 2000), proteasome activity is required for the clearance of polyQ-expanded AR as well (Bailey et al., 2002; Nath et al., 2018; Arnold et al., 2019). In addition, a role for the proteasome was observed in the nuclear inclusion-associated proteolysis of polyQ-expanded AR; this role appeared late in the maturation of nuclear inclusions, suggesting that AR proteolysis within inclusions likely does not contribute to toxicity (Heine et al., 2015). Nonetheless, this observation of nuclear mutant AR proteolysis indicates that proteasomes can act on polyQ-expanded AR, albeit inefficiently, within the nuclear compartment. Although proteasomes are found in both the nuclear and cytoplasmic subcellular compartments, the relative localization may vary by cell type and cell cycle stage (Wojcik and DeMartino, 2003; Kito et al., 2020; Guo, 2022). Moreover, not only are catalytically active proteasomes primarily found in the cytoplasm (Dang et al., 2016), but many nuclear proteins require export to the cytoplasm for their proteasomal degradation (Chen and Madura, 2014), demonstrating the importance of cytoplasmic proteasomes in cellular protein degradation.

Despite the demonstrated role of cytoplasmic proteasomes in the degradation of many nuclear proteins, including polyQ-expanded AR, a role for nuclear proteasomes in the degradation of misfolded proteins has been demonstrated (Prasad et al., 2010; Park et al., 2013). Moreover, recent evidence indicates that nuclear AR can be efficiently degraded by nuclear proteasomes in the absence of androgens in models of prostate cancer (Lv et al., 2021). Despite this finding of efficient nuclear proteasomal degradation of AR in prostate cancer models, the observation that the inefficient proteasomal degradation of polyQ-AR contributes to its aggregation (Heine et al., 2015), and that enhancing nuclear export of polyQ-AR substantially enhances its proteasomal degradation (Arnold et al., 2019), suggests that polyQ-expanded AR is more efficiently degraded by proteasomes within the cytoplasmic compartment. The role of hormone binding and release and the mechanisms that impact the differences in efficiency between nuclear and cytoplasmic proteasomal degradation of polyQ-expanded AR remain to be determined.

A role for AR ubiquitination in the pathogenesis of SBMA derives from the finding that the DUB USP7 is required for disease manifestations *in vitro* and *in vivo* (Pluciennik et al., 2021). USP7 was found to preferentially interact with polyQ-expanded AR and deubiquitinate it at 8 specific lysine residues, K17, K222, K718, K826, K837, K862, K911, and K913. USP7 depletion decreased mutant AR aggregation and toxicity in cell models and in both transgenic *Drosophila* and mouse SBMA models (Pluciennik et al., 2021). Additionally, USP7 preferentially interacted with soluble, monomeric, and aggregated toxicity-associated species of polyQ-expanded AR (Berger et al., 2015; Heine et al., 2015; Pluciennik et al., 2021). USP7 knockdown reduced the level of this aggregated species as well as of a population of monomeric AR. Mutation of one of the eight USP7-regulated ubiquitination sites (K17) substantially increased polyQ-expanded AR aggregation, suggesting the importance of this site for mutant AR homeostasis. USP7 also interacted with polyQ-expanded mHtt and polyQ-expanded Ataxin-3 and was shown to attenuate pathology induced by polyQ-expanded Ataxin-3 (Pluciennik et al., 2021).

The U-Box E3 ligase CHIP polyubiquitinates both WT and polyQ-expanded AR; however, its overexpression preferentially ubiquitinated and cleared polyQ-expanded AR, leading to phenotypic improvements in cultured neurons and transgenic polyQ-expanded AR mouse models of SBMA (Adachi et al., 2007). Like the DUB USP7 (Pluciennik et al., 2021), CHIP also interacts with mHtt and polyQ-expanded Ataxin-3 (Jana et al., 2005), and its overexpression increased the ubiquitination and degradation of other polyQ-expanded proteins such as mHtt, mutant Ataxin-1, and mutant Ataxin-3 (Jana et al., 2005; Miller, 2005; Al-Ramahi et al., 2006; Williams et al., 2009). CHIP-mediated ubiquitination is dependent on the Hsp90/Hsp70 chaperone machinery (Wang et al., 2010). Indeed, both the ubiquitinating U-box domain and the chaperone protein-interacting TPR domain facilitate CHIP’s ubiquitinating ability.

The regulation of AR ubiquitination by PTMs leads to potentially tractable therapeutic approaches. For example, the Akt pathway promotes phosphorylation of AR at S215 and S792. Overexpression of insulin-like growth factor (IGF-1) activated Akt in skeletal muscle of AR97Q transgenic mice, resulting in increased polyQ-AR phosphorylation at S215 and its increased proteasomal degradation. PolyQ-expanded AR aggregation was reduced, motor defects were attenuated, onset of disease was delayed, and mouse survival was extended (Palazzolo et al., 2007, 2009; Rinaldi et al., 2012). In contrast, phosphorylation at S96 increased polyQ-expanded AR aggregation and toxicity; its inhibition lowered levels of polyQ-expanded AR in a proteasome-dependent manner, and ameliorated SBMA phenotypes in cell and knock-in mouse models (Polanco et al., 2016).

## The role of additional ubiquitin-proteasome system components in spinal and bulbar muscular atrophy

Nrf1 represents an important transcriptional regulator of proteasome subunit expression during UPS impairment or proteasome stress, thereby regulating proteasome homeostasis (Radhakrishnan et al., 2010; Sha and Goldberg, 2014; Lehrbach et al., 2019). Nrf1 is itself regulated by its proteolytic cleavage at the ER by DDI2, thus enabling its nuclear translocation for transactivation (Koizumi et al., 2016; Lehrbach and Ruvkun, 2016; Northrop et al., 2020). Both Nrf1 and DDI2 levels were reduced in skeletal muscle of a knock-in mouse model of SBMA (Nath et al., 2018). Importantly, activation of Nrf1 not only increased the levels of many proteasome subunits, including 20S CP, 19S RP, and 11S RP components, it also increased the ubiquitination of polyQ-expanded AR and mediated its proteasomal degradation in SBMA patient fibroblasts and in *Drosophila* and mouse models of SBMA (Bott et al., 2016), indicating a therapeutic approach to enhance mutant AR degradation.

The predominantly nuclear 11S proteasome activator REGγ was observed to impact polyQ-expanded AR in a cell-specific manner. In a PC12 model of SBMA, REGγ enhanced polyQ-expanded AR aggregation and cell toxicity in manner that was independent of its ability to cap the 20S proteasome and correlated with its inhibition of MDM2-mediated polyubiquitination of polyQ-expanded AR. However, REGγ had a distinct effect in cultured SBMA mouse motor neurons, rescuing cellular toxicity in a proteasome binding-dependent manner (Yersak et al., 2017).

Components of the ubiquitination cascade are altered in muscles of SBMA mouse models. For example, the E2 ubiquitin conjugating enzyme UBE2Q1 was upregulated (Rusmini et al., 2015) and the E2 enzyme Ube2T was suppressed in disease-relevant skeletal muscle of SBMA knock-in mice (Nath et al., 2018). Although several muscle-specific E3 ligases are known to be induced during skeletal muscle atrophy (Bodine et al., 2001; Sartori et al., 2013; Bodine and Baehr, 2014; Milan et al., 2015; Baehr et al., 2022), the alterations in SBMA mouse muscle indicate the activation of a UPS pathway that is distinct from that observed upon general skeletal muscle atrophy (Rusmini et al., 2015; Nath et al., 2020; Forouhan et al., 2022). In addition to these changes, several UPS-related genes were reduced in diseased tissues of SBMA mouse models, including both core and cap proteasomal subunits and E2 ubiquitin conjugating enzymes (Tokui et al., 2009; Nath et al., 2018). In contrast, elevated proteasome activity was observed in quadriceps muscle of the same knock-in mouse model of SBMA (Rocchi et al., 2016), perhaps indicating a compensatory mechanism. The importance of these changes in disease manifestation is suggested by the observation that either surgical castration or skeletal muscle-specific AR ASO treatment rescued the expression of many of these UPS genes, concomitant with a restoration of motor function (Nath et al., 2018).

Despite alterations in the expression of some UPS components, the evidence from the above studies does not indicate the presence of a global, organism-wide impairment of the UPS in SBMA. Rather, the changes in proteasomal expression and activity in SBMA appear in a tissue-specific manner, with greater changes in skeletal muscle than in neuronal tissues. Additionally, the pathways impacting muscular atrophy appear to be distinct from the known skeletal muscle atrophy profile. Regardless, the above studies demonstrate that altering various components of the UPS can result in attenuation of the disease phenotype in SBMA model systems and suggest that the modulating UPS components could be a viable approach to developing disease treatment.

## Research gaps and points of consideration in the development of ubiquitin-proteasome system-dependent therapeutics for spinal and bulbar muscular atrophy

Several studies have evaluated the role of E3 ligases, DUBs, or other UPS components in disease pathogenesis. Regulation of protein degradation by the UPS can be manipulated to promote clearance of misfolded and pathogenic proteins and rescue the phenotypes in disease models. Such an approach represents a potentially powerful one for the treatment of SBMA and other polyQ disorders. New selective ubiquitination-based technologies such as proteolysis-targeting chimeras (PROTACs) and selective AR degraders (SARDs) are being developed for treating AR-related disorders by the targeted degradation of AR (Auvin et al., 2019; Beretta and Zaffaroni, 2019; Hwang et al., 2019; Ponnusamy et al., 2019; Alabi and Crews, 2021; Zeng et al., 2021; Hu and Crews, 2022).

Due to the keen interest in this therapeutic area, some important points may be considered during the development of therapeutics that rely on the UPS. First, target selection is of primary importance. Some components of the UPS facilitate AR transcriptional activity or impact its intracellular levels. Due to the vast number of AR target genes and their functions, it may be desirable to preserve AR function during treatment to ensure overall improved quality of life for patients. Similarly, while some UPS components clearly contribute to SBMA pathogenesis, these proteins may also serve important functions independently of the AR and their depletion could have unintended consequences on health. Depletion of E3 ligase RACK1 rescued aggregation and/or cell toxicity in *Drosophila* models of polyQ disease (Xie et al., 2021), but overexpression of E3 ligases WWP1 and TRAF6 increased mHtt aggregation (Zucchelli et al., 2011; Lin et al., 2016). Indeed, one E3 ligase acts on Htt in two very different ways. K48-polyubiquitination by Ube3a mediated degradation of Htt; however, K63-polyubiquitination by the same E3 ligase promoted Htt aggregation (Bhat et al., 2014).

Additionally, the specific levels of UPS components such as E3 ligases and DUBs may differentially impact mono- and polyubiquitination or have distinct effects on substrate proteins. For example, the E3 ligase MDM2 regulates its substrate protein, p53, in two ways. Low levels of MDM2 promote p53 monoubiquitination and nuclear export, while higher levels promote p53 polyubiquitination and proteasomal degradation in the nucleus (Li et al., 2003). A partial knockdown of the DUB USP7 in a cellular model of SBMA rescued AR-associated aggregation and cell death; yet, a near-complete knockdown failed to do so (Pluciennik et al., 2021), likely due to confounding effects on other proteins that impact AR levels.

The functional redundancy of E3 ligases is another aspect to consider. Different E3 ligases are capable of acting on and ubiquitinating the same substrate protein. Thus, another E3 ligase may compensate for a depleted one. This phenomenon has been observed in plants (Feke et al., 2019), yeast (Breckel and Hochstrasser, 2021), and also specifically in the context of ubiquitinating polyQ-expanded disease proteins (Morishima et al., 2008). This is a key aspect of the role of the E3 ligase in preserving cellular proteostasis. However, it adds another challenge for suppressing the activity of E3 ligases and must inform the research and development of therapeutics targeting proteins related to ubiquitination and the UPS (Garcia-Barcena et al., 2020).

## Author contributions

MS, AP, and DM developed the structure of the manuscript. MS wrote the initial drafts of the manuscript. AP and DM edited and contributed to the writing of the final version of the manuscript. All authors approved the submitted version of the manuscript.
